# Post-COVID-19 Vaccination Infection Among Adults in Saudi Arabia: A Cross-Sectional Study

**DOI:** 10.7759/cureus.47552

**Published:** 2023-10-23

**Authors:** Rehab A Mohammed, Omar Baqais, Samaher G Basalib, Abdulaziz A Owaidah, Abdulrahman Mirza, Randa M Alharizi, Intessar Sultan

**Affiliations:** 1 Department of Internal Medicine, Faculty of Medicine for Girls, Al-Azhar University, Cairo, EGY; 2 Department of Medicine, Ibn Sina National College for Medical Studies, Jeddah, SAU; 3 Department of Internal Medicine, Faculty of Medicine, Cairo University, Cairo, EGY

**Keywords:** astrazeneca vaccine, pfizer vaccine, breakthrough infection, vaccination, covid-19

## Abstract

Background: While vaccines were one of the most effective tools to combat the COVID-19 pandemic, breakthrough infections have been reported.

Aim of the work: We aim to evaluate the effectiveness of Pfizer and AstraZeneca vaccines in preventing breakthrough infection, as well as to determine the possible risk factors and outcomes of post-vaccination infection.

Methods: This is a cross-sectional study using self-reported data of adult Saudi residents, including Saudi and non-Saudi people who received at least two doses of either Pfizer or AstraZeneca vaccines. Based on the presence of COVID-19 symptoms that were confirmed by PCR, the participants were classified into three groups: (1) those with evidence of infection before vaccination, (2) those who had infection after vaccination, and (3) those who had infection before and after vaccination. For further evaluation, we compared the severity and outcomes in the participants who were infected before and after vaccination.

Results: The study included 694 participants: 69.1% received three doses of the vaccine, and 71.1% of them were vaccinated with the Pfizer vaccine. COVID-19 infection was reported in 48.3% of the total subjects, with a higher infection rate (17.8%) after vaccination compared to 12.5% before vaccination. Additionally, 18.32% of participants experienced infection both before and after vaccination. Out of the total 694 participants, 137 (19.7%) had breakthrough infections. Pfizer vaccine was more prevalent among the non-infected group (74.25% vs. 65.5%), while AstraZeneca vaccine was more prevalent among the infected group (6.4% vs. 5.9% (p<0.039). Diabetes was significantly higher among the infected group (16.9% vs. 8.1%, p=0.001, OR=2.29, 95% CI=1.42-3.68). Among those who were infected before and after vaccination, 71.9% reported less severe symptoms after vaccination.

Conclusion: Breakthrough infections may occur after vaccination; however, vaccines are overall effective in preventing severe symptoms. Pfizer vaccine appeared to be more effective in preventing COVID-19 infection. The presence of comorbidities, including diabetes, may increase the risk of infection.

## Introduction

The approval of SARS-CoV-2 vaccines for public health use in December 2020 was seen as one of the most successful strategies to reduce the impact of the COVID-19 outbreak and possibly ending the pandemic [1].

Most COVID-19 vaccines are intended to induce immunological responses through the production of neutralizing IgG and IgA antibodies against the SARS-CoV-2 spike protein. In addition to systemic immunity, mucosal immune responses are important in reducing the viral spread [2]. Suppression of SARS-CoV-2 transmission can be achieved by blockage of viral entry in mucosal cells of the oral cavity and pharynx through mediation of the mucosa-associated lymphoid tissue (MALT) [3].

However, this protective effect of vaccines wanes with time, and the antibody levels decrease substantially within a few months after vaccination. Many cases of post-vaccination infections have been reported after vaccination. Viral gene sequencing has found that some of these patients were infected by different strains [4]. There are a few studies of COVID-19 infection and related risk factors among vaccinated individuals from Saudi Arabia [5].

Characterizing people at a greater risk of breakthrough infections, hospitalization, or death is critical to promote targeted interventions and to enhance the protection of vulnerable populations [6]. Therefore, we aimed to evaluate COVID-19 infection post-vaccination and its associated factors among vaccinated COVID-19 cases in adult Saudi Arabian residents.

## Materials and methods

Study design and participants

This was a cross-sectional community-based study among residents in Saudi Arabia (including Saudi and non-Saudi) from July to November 2022 to investigate the occurrence of COVID-19 infection among vaccinated people. Inclusion criteria included vaccinated adults who had received at least two doses of either Pfizer or AstraZeneca vaccines, had a good understanding of Arabic or English languages, were willing to participate in the study, were willing to complete the survey, and were willing to provide informed consent. To compare the disease profile of COVID-19 infection before and after vaccination, we classified the participants into three groups: (1) those with evidence of infection before vaccination, (2) those who had infection after vaccination, and (3) those who had infection before and after vaccination. We considered infection 14 days after full primary vaccination as a breakthrough infection as defined by the Centers for Disease Control and Prevention (CDC) [7]. Individuals who experienced symptoms but did not undergo PCR confirmation were excluded from our study.

Sample size calculation

A sample size of 384 using Epi-Info (version 7; Centers for Disease Control and Prevention, Atlanta, Georgia) is desired based on the 5% margin of error, a design effect of 1, a cluster of 1, and an expected frequency of 50%, but a final sample of 694 was collected. Participants were selected using a non-probability convenient sampling technique.

Data collection and tools

The collection of data was based on an online Google Forms questionnaire. The questionnaire was constructed from the Saudi FDA and Centers for Disease Control and Prevention (CDC) official websites that are used for reporting COVID-19-related side effects, including COVID-19 infection [8] with some modifications. The questionnaire was constructed in both Arabic and English languages. The questionnaires inquired facts about their socio-demographics, their current medical conditions, the type and number of doses of vaccine received, and if they had a positive COVID-19 PCR test before or after the vaccination. Those who were infected before and after vaccination were asked to compare the severity of COVID-19 symptoms using a scale from 1 to 10.

Ethical consideration

Ethics and confidentiality of the data were assured. The study received approval from the Ibn Sina National College Institutional Research Review Board (IRB-09-04082022).

Statistical analysis

Statistical Product and Service Solutions (SPSS) (version 22; IBM SPSS Statistics for Windows, Armonk, NY) was used to analyze the data and to construct the figure. Descriptive analysis was presented as frequency and percentage. A chi-squared test was used to compare those with and without COVID-19 infection after vaccination with an estimation of the odds ratio, when possible, with a 95% confidence interval (95% CI). The level of significance for the study was set at < 0.05.

## Results

A total of 694 participants were enrolled in this study. The demographic characteristics of the participants are shown in Table 1; the median age of the participants was 46 years (interquartile range=36-54). More than half of the participants (56.5%) were women, most of the participants (84.6%) were from Saudi, 28.7% were healthcare workers, and 49% had chronic illnesses. Most of them (69.1%) received three doses of the vaccine, most of them (71.1%) were vaccinated with the Pfizer vaccine, and about 23% received a mixed vaccination regimen of both AstraZeneca and Pfizer vaccines (Table 2).

**Table 1:** Demographic and clinical characteristics of the participants HCW: Healthcare worker, CVD: Cardiovascular diseases, COPD: Chronic obstructive pulmonary disease

		Count 694	Column N %
age	<18	63	9.1%
	18-30	442	63.7%
	31-50	136	19.6%
	>50	53	7.6%
Gender	Male	302	43.5%
	Female	392	56.5%
Nationality	Non-Saudi	107	14.4%
	Saudi	587	84.6%
Work	NOT HCW	495	71.3%
	HCW	199	28.7%
Smoking	NO	583	84.0%
	YES	111	16.0%
Comorbidities	NO	353	50.9%
	Liver disease	6	0.9%
	Allergy	35	5.0%
	CVD	39	5.6%
	Diabetes	73	10.5%
	Autoimmune	4	0.6%
	Obesity	46	6.6%
	COPD	46	6.6%
	Thyroid	18	2.6%
	Sickle	17	2.4%

**Table 2:** Vaccination and COVID-19 infection data

		Count (694)	Column N %
Number of doses	Two doses	215	30.9%
	Three or more doses	479	69.1%
Vaccine type	Pfizer	493	71.1%
	Astra	43	6.1%
	Mixed	158	22.9%
History of COVID-19 infection	No infection	358	51.7%
	Infection before vaccination	86	12.3%
	Infection after vaccination	124	17.8%
	Infection before and after vaccination	126	18.2%
Severity of COVID-19 symptoms	Mild	210	30.2%
	Moderate	458	65.9%
	Severe	26	3.7%
History of post-vaccination infection total no. (250)	After the 2^nd^ dose	103	41.2%
	After the 3^rd^ dose	147	58.8%

**Figure 1:**
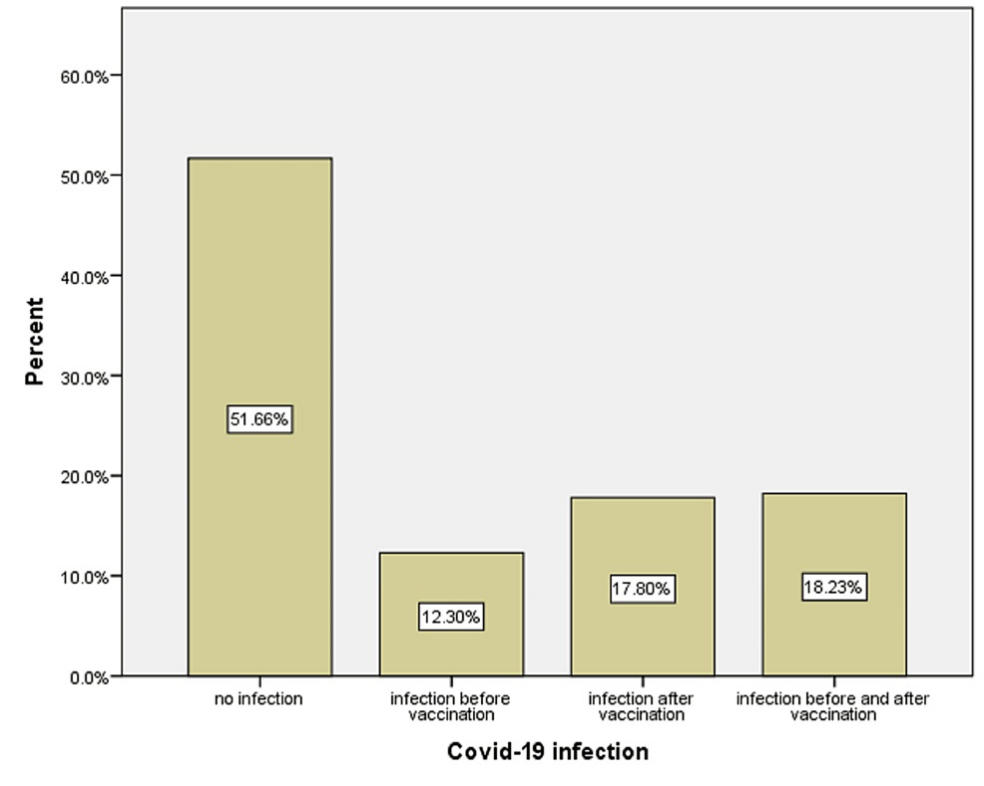
The percentage of infection before and after vaccination

We also investigated and compared the infection rates before and after the vaccination. There was a history of PCR-positive COVID-19 infections in about 48.3%of the total subjects, with a higher infection rate (17.8%) after vaccination compared to the 12.5% infection rate before vaccination, while 18.32% had a history of COVID-19 infection before and after vaccination (Figure 1).

More than 95% of the participants categorized their COVID-19 symptoms as mild to moderate, with only 3.7% having severe symptoms needing medical consultation. However, no one reported hospital admission (Table 2).

Table 3 shows the comparison between those with and without infections after vaccination concerning their characteristic data. There were no significant differences regarding their age, gender, and smoking status. However, comorbidities were significantly prevalent among participants who had COVID-19 infection after vaccination (58.6% vs. 43.4%, p<0.001, OR=1.85, 95% CI=1.35-2.53). Additionally, diabetes was significantly higher in the same group (16.9% vs. 8.1%, p=0.001, OR=2.29, 95% CI=1.42-3.68).

**Table 3:** Comparison between those with and without COVID-19 infection after vaccination concerning their characteristic data

		No COVID-19 infection after vaccination N=444 (64%)		COVID-19 infection after vaccination N=250 (36%)		p	Odds ratio	95% CI	
		N	%	N	%			Upper limit	Lower limit
Age group	<18	45	10.2%	16	6.4%	0.346	--	--	--
	18-30	281	63.6%	161	64.7%				
	31-50	82	18.6%	53	21.3%				
	>50	34	7.7%	19	7.6%				
Gender	Males	191	43.2%	109	43.8%	0.886	.977	.715	1.337
	Females	251	56.8%	140	56.2%				
Health care working	No	317	71.7%	175	70.3%	0.689	1.072	.762	1.509
	Yes	125	28.3%	74	29.7%				
Smoking	NO	378	85.5%	202	81.1%	0.131	1.374	.909	2.078
	YES	64	14.5%	47	18.9%				
Comorbidities	No	250	56.6%	103	41.4%	<0.001	1.85	1.35	2.53
	Yes	192	43.4%	146	58.6%				
Diabetes	NO	406	91.9%	207	83.1%	0.001	2.29	1.42	3.68
	YES	36	8.1%	42	16.9%				
Cardiac disease	No	418	94.6%	235	94.4%	0.915	1.038	0.527	2.044
	Yes	24	5.4%	14	5.6%				
COPD	No	416	94.1%	229	92.0%	0.276	1.397	0.763	2.559
	Yes	26	5.9%	20	8.0%				
Obesity	No	413	93.4%	233	93.6%	0.945	0.978	0.520	1.838
	Yes	29	6.6%	16	6.4%				

In Table 4, we compared both groups regarding the type of received vaccine and the number of doses. The Pfizer vaccine was more prevalent among the non-infected group (74.25% vs. 65.5%), while AstraZeneca and mixed vaccination were more prevalent among the infected group (6.4% vs. 5.9% and 28.1% vs. 19.9%, respectively; p<0.039). Receiving three or more doses was more prevalent among the infected compared to the non-infected group (73.9% vs. 64.5%; p=0.008).

**Table 4:** Comparison of vaccination data between those with and without COVID-19 infection after vaccination

		No COVID-19 infection after vaccination N=444		COVID-19 infection after vaccination N=250		P
		N	%	N	%	
Type of vaccine	Pfizer	329	74.2%	163	65.5%	0.039
	AstraZeneca	27	6 %	16	6.4%	
	Mixed	88	19.9%	70	28.1%	
Number of doses	Two doses	159	35.8%	66	26.4%	0.008
	>= 3 doses	285	64.1%	184	73.6%	

In Table 5, we analyzed the profile of the COVID-19 infection after vaccination (n=250). More than half (55%) of the participants reported a positive COVID-19 test more than 14 days after vaccination, 11.6% had an infection 7-14 days after vaccination, and 33.4% could not remember the time of onset of infection. In about 57% of the participants, the symptoms lasted less than three days, while the symptoms extended more than seven days in 18.5%. It was observed that 126 participants had COVID-19 infection before and after vaccination, 71.9% of them reported more severe symptoms before vaccination, and only 28.1% experienced more severe symptoms after vaccination. However, no history of hospital admission in both groups.

**Table 5:** Characteristics of COVID-19 infection after vaccination

		COVID-19 infection after vaccination N=250	
		N	%
Onset of infection after vaccination	< 14 days	30	11.6%
	> 14 days	137	55.0%
	Don't know	83	33.4%
Duration of COVID-19 symptoms	< 3 days	142	57%
	3-7 day	62	24.9%
	> 7 days	46	18.5%
Having more severe symptoms* total no. (126)	After getting the COVID-19 vaccines	40	28.1%
	Before getting the vaccines	86	71.9.%
*Infection before and after vaccination			

## Discussion

In this community-based study, while 69%% of the participants received three doses of COVID-19 vaccines, around 48% of them had COVID-19 infection with a higher infection rate after vaccination (17.8%) compared to 12.5% before vaccination. This pattern of an increasing infection rate after vaccination was observed in many countries, including Chile, Hungary, UAE, Qatar, and Serbia [9].

These results may be explained by the immaturity of immune response in the early period after vaccination, and a lower rate may be expected later after the development of immune response [9]. Another explanation is that the presence of systemic reactions after vaccination can enhance testing for COVID-19 in the first few days after vaccination [10].

The CDC defined the infections occurring two weeks after the full vaccination as "breakthrough infections" [7]. Accordingly, 137 (19.7%) out of the total 694 participants had breakthrough infections in our study. This is comparable to the results of Walsh et al. [11], who found that 25% of healthcare workers had breakthrough infections and their higher incidence may be due to the environment of the participants since healthcare workers are more susceptible to infections.

Another finding in our study is that the vaccine breakthrough infection appeared to be more prevalent after the third dose, suggesting that booster doses may be less protective against COVID-19 variants. Higher booster coverage rates and non-pharmaceutical interventions may be needed to achieve public benefit and to slow the spread of COVID-19 variants [12].

A higher infection rate was more prevalent in the AstraZeneca and mixed regimens compared to the Pfizer vaccine. This result agrees with those of Almufty et al. [13], who found that the Oxford-AstraZeneca vaccine appeared to be less effective than the Pfizer vaccine in preventing SARS-CoV-2 infection. Another study conducted by Sheikh et al. [14] found no statistical differences after vaccination in participants who received an adenovirus vector vaccine and those who received an mRNA vaccine.

Despite higher COVID-19 infection post-vaccination in our study, the severity of symptoms was less after vaccination. This is in line with the results of Ayoubkhani et al. [15] who advocate that the COVID-19 vaccination is effective in preventing severe outcomes. 

In this study, the presence of comorbidities including diabetes was associated with a higher infection rate after vaccination. This observation agrees with that of Dagan et al. [16] who studied the effect of comorbidities on serostatus post-COVID-19 vaccination, and they found that the presence of a comorbid condition was associated with seronegative status. Lower vaccination response in diabetics may be due to the chronic systemic low-grade inflammation associated with diabetes that may lead to impaired macrophage activation, defective leukocyte recruitment, and lower production and function of antibodies [17].

Limitations

Our study is observational, so causality cannot be deduced. Some people may have the coronavirus and never show symptoms or may develop mild symptoms. Such people may deny their infection status or may not request the test, so the assessment based on self-reported symptoms and or self-requested PCR testing is another limitation of our study. However, many studies used self-reported data for assessment of the COVID symptoms after vaccination. Because our study depends on each respondent's response and the regions where the survey is dispersed, the data do not accurately represent the epidemiological condition [18,19].

## Conclusions

Although we found a slightly increased infection rate after vaccination, the severity of symptoms was significantly less after vaccination. Breakthrough infections may occur in fully vaccinated individuals, who may in turn spread the virus to others. These findings may highlight the need to develop guidelines by public health organizations throughout the world, thereby preventing possible future outbreaks. The Pfizer vaccine appeared to be more effective in preventing COVID-19 infection. Studies are needed to understand the mechanisms and the causal relationships of infection after vaccination.
